# The impact of canine-assisted intervention on stress reduction among university students in Thailand

**DOI:** 10.1371/journal.pone.0318777

**Published:** 2025-03-12

**Authors:** Saengduean Yotanyamaneewong, Daranee Junla, Janine L. Brown, Nathida Siriapaipant, Naruedee Yodkamol, Worapat Prachasilchai, Adul Saengthong, Pratch Sanguansak, Thanapun Kankonsue, Veerasak Punyapornwithaya, Chalutwan Sansamur, Korakot Nganvongpanit, Jaruwan Khonmee

**Affiliations:** 1 Department of Psychology, Faculty of Humanities, Chiang Mai University, Chiang Mai, Thailand; 2 Smithsonian National Zoo & Conservation Biology Institute, Front Royal, Virginia, USA; 3 Faculty of Veterinary Medicine, Chiang Mai University, Chiang Mai, Thailand; 4 Chiang Mai University Library, Chiang Mai University, Chiang Mai, Thailand; 5 Research Center for Veterinary Biosciences and Veterinary Public Health, Faculty of Veterinary Medicine, Chiang Mai University, Chiang Mai, Thailand; 6 Akkhraratchakumari Veterinary College, Walailak University, Nakhon Si Thammarat, Thailand; University of Mississippi School of Pharmacy, UNITED STATES OF AMERICA

## Abstract

Stress negatively impacts university students, leading to adverse outcomes. While canine-assisted intervention (CAI) has been shown to reduce self-reported stress, no studies have investigated stress levels and associated biomarkers in dogs and students simultaneously. This study examined salivary cortisol, blood pressure, and pulse rate in 122 university students experiencing self-reported moderate to high stress before an encounter with a dog (T1), immediately before meeting a dog (T2), and after spending 15 minutes interacting with a dog (T3). Participants assessed their stress level using a visual analog scale, and blood pressure and pulse rate were measured at three time points. Salivary cortisol was also measured at T1 and T3. Six privately owned dogs, all in good health and comfortable with strangers, participated in the intervention sessions. Salivary and fecal cortisol samples from the dogs were collected in the morning before commencing activities, at noon, and in the evening after human interactions ended. The results showed that the expectation of interacting with dogs reduces self-reported stress, pulse rate, and salivary cortisol, which remained significantly lower after the interaction. Salivary cortisol concentrations in dogs did not differ throughout the day. By contrast, fecal glucocorticoid metabolite concentrations during the week dogs interacted with students were higher (P =  0.0012) than those during the week post-experiment, which, based on behavior, appeared to indicate positive stimulation. These findings highlight the potential of integrating CAI into university stress management programs. Future research could explore extending these benefits to community dogs, long-term effects, and enhancing accessibility to this form of stress relief.

## Introduction

Stress is a state of mental tension or concern arising from challenging circumstances and is a natural human reaction that motivates individuals to confront obstacles and perceived threats [1]. Its presence is universal, albeit varying in intensity among individuals [2]. Although essential to life and normal physiological functioning, reactions to stress can significantly influence holistic health and welfare, both positively and negatively [3]. Stress is a prevalent occurrence among university students, with incidents varying but remaining notably high. For instance, 46% of 5,092 young adults were reported to have experienced a psychiatric disorder [4], while 33% of 80,121 students across 106 institutions exhibited stress related to their academic pursuits [5]. A study involving 822 undergraduate and graduate students at a private university found that 23% were diagnosed with mental illness stemming from stress [6]. The stress experienced by university students can lead to non-graduation or negative outcomes [7]. Additionally, stress has been linked to physical ailments such as headaches or muscle pain [8–11], suicidal ideation, and attempts at self-harm. A study conducted in 2016 revealed that 29% of 5,572 university students from 12 countries reported having contemplated suicide, with 7% reporting actual attempts [12].

Numerous interventions have been implemented to alleviate stress among university students, including cognitive–behavioral therapy, coping skills development, social support interventions, relaxation training, mindfulness-based stress reduction, and psychoeducation [13]. Another approach focuses on pet therapy, specifically interactions with dogs [14], which has been shown to have stress-reducing effects [15–17]. Those studies provided 10–15 minutes of interaction between students and dogs, resulting in self-reported decreases in stress levels. Canine-Assisted Intervention (CAI) is easy to implement and can effectively relieve momentary stress in university students (e.g., during exams) [18,19]. Our study employed a structured CAI protocol with a specific goal of stress reduction, involving collaboration with psychologists and veterinarians, but using non-certified dogs without the presence of the handler. This protocol featured hands-on interactions between students and dogs using self-report stress and cortisol as primary stress metrics, alongside measures of autonomic nervous system cardiac activity (blood pressure, pulse rate) for a comprehensive evaluation. Cortisol is well-established as a reliable, non-invasive stress indicator in both humans [20,21] and animals [22], making it suitable for testing the effects of CAI on university students. Moreover, few studies have measured both self-reported and salivary cortisol outcomes from CAI. We hypothesized that stress, salivary cortisol concentrations and cardiac function measures would be lower in students following 15-minute physical interactions with dogs. We also measured salivary cortisol and fecal glucocorticoid metabolites (fGCM) in the dogs to determine if interacting with students affected adrenal activity over the short or long term. This study represents the first time CAI has been tested and evaluated in Thailand.

## Materials and methods

### Study populations

Approvals were obtained by the Chiang Mai University Research Ethics Committee, Thailand (CMUREC 66/233) for the human study and The Animal Care and Use Committee at the Faculty of Veterinary Medicine, Chiang Mai University (R1/2567) for the animal study. Students were enrolled at Chiang Mai University (Chiang Mai, Thailand) between January 8–12, 2024. Participants were included if they had a score of 14 or above (out of 40) on the Perceived Stress Scale (PSS-10) [23]. The sample size was calculated using g * power and set at a minimum of 110 individuals, including a dropout rate of 15% [24]. The calculation was based on an alpha level of 0.05, and a power level of 0.80. Participants were selected through purposive sampling and met specific criteria: 1) were a university student at the undergraduate level; 2) being 18 years of age or older; 3) had PSS scores indicating moderate to high-stress levels (14 or above); and 4) voluntarily participated. All students were over 18 years of age and signed, in the presence of two researchers, a written consent form that was explained verbally before signing. Students were excluded if they reported a fear, dislike, or allergy to dogs. The study population comprised 122 individuals, including 30 males and 92 females.

Six private-owned dogs participated in the intervention sessions and were selected because they 1) exhibited good health and had completed a vaccination program, 2) were comfortable with human interactions since a young age, 3) displayed an affinity for human interaction, and 4) demonstrated no fear of strangers. The dogs were between 3 and 6 years old, comprising males and females. Five chihuahuas were raised by veterinarians, while one Shetland sheepdog was raised by a psychologist. All dogs were non-certified and participated in Canine-Assisted Interventions (CAI) for the first time, having received approximately 1 week of preparation prior to the research. Additionally, the dogs participated without the presence of the owner. The intervention process was supervised by veterinarians and psychologists.

### Data and sample collection

#### University students.

**Fig 1:**
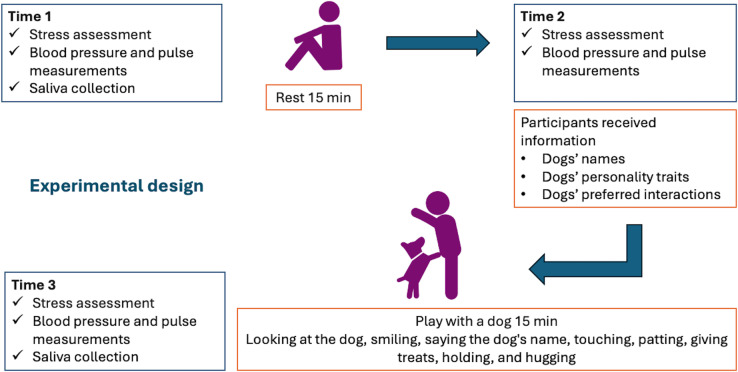
Experimental design for the human component of the study. Schematic of the experimental design of the study at Time 1 (T1), Time 2 (T2), and Time 3 (T3).

The project was conducted over a 5-day period in the main library of Chiang Mai University. Data and samples were collected from students before and after playing with a dog (Fig 1). Participants first completed personal information questionnaires (sex, academic year, and faculty affiliation) upon enrollment and then underwent a stress assessment utilizing a single-item visual analog scale (VAS) that has been employed in mental health research to determine a participant’s self-rating of psychological constructs, including happiness, depression, and stress [25–28]. Using the VAS, participants rated their stress on a 7-point scale (1 =  minimal stress, 7 =  extreme stress) at Time 1 (T1), Time 2 (T2), and Time 3 (T3) (Fig 1). Systolic (SBP) and diastolic (DBP) blood pressure and pulse rates (PR) (Omron, Thailand) also were taken. Saliva was then collected using Salivette® kits (Sarstedt, AG and Co, Numbrecht, Germany) by swiping the absorbent swab inside the buccal area for 30–60 seconds, which took less than 5 minutes to complete. At T1, the students completed all three measures: VAS stress assessment, BP, and PR, and had saliva collected, followed by a 15-minute rest period. At T2, students completed a second stress assessment with BP and PR measurements. Following this, the student interacted with one dog for 15 minutes. After that, at T3, the participants underwent a third stress assessment, BP and PR measurements, and a second saliva collection. All participants completed all measurements at every time point.

### Intervention dogs

**Fig 2:**
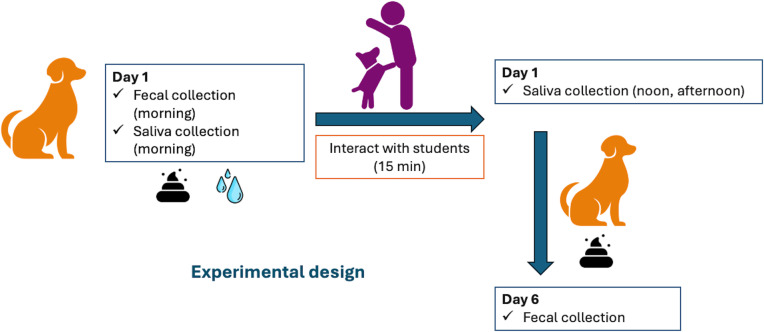
Experimental design for the canine component of the study. Schematic of the experimental design of the study at Time 1 (T1), Time 2 (T2), and Time 3 (T3).

Saliva samples were collected from each dog three times a day: morning (before commencing activities at around 1000 hr), noon, and evening (after human interactions ended at around 1600 hr). Each dog interacted with approximately three students each day for 15 minutes during the testing week. The behavior of the dogs was monitored subjectively before inviting the students in and throughout the day to ensure they remained comfortable. Fecal samples were collected from the dogs each morning during the study and for an additional 5 days after the conclusion of the experiment to measure fGCM concentrations (Fig 2).

### Salivary cortisol analysis

Saliva (students and dogs) and fecal samples (dogs) were kept in 4°C coolers for less than 8 hr and then frozen at ‒20°C. Samples were centrifuged at 1500 x g for 5 min at 15°C and stored frozen until analyzed for cortisol using a double-antibody enzyme immunoassay (EIA) with a polyclonal rabbit anti-cortisol antibody (R4866) [29]. Second antibody-coated plates were prepared by adding 150 µl of anti-rabbit IgG (0.01 mg/ml) to each well of a 96-well microtiter plate (Nunc-Immuno Maxisorp, Thermo Fisher Scientific, Roskilde, Denmark) and incubated at room temperature (RT) for 15–24 hr. The wells were then emptied and blotted dry, followed by adding 250 µ L blocking solution (100 mM phosphate, 150 mM sodium chloride, 1% Tween 20, 0.09% sodium azide, 10% sucrose, pH 7.5) and incubating for 15–24 hr at RT. After incubation, all wells were emptied, blotted, and dried at RT in a desiccating cabinet (Sanpla Dry Keeper, Sanplatec Corp., Auto A-3, Japan) with loose desiccant in the bottom. After drying (humidity <  20%), plates were heat-sealed in a foil bag with a 1 g desiccant packet and stored at 4°C until use.

Plates were brought to RT and saliva samples (50 µ L, diluted 1:5 for students and 1:2 for dogs) or cortisol standards (50 µl) were added to appropriate wells. Cortisol-horseradish peroxidase (HRP) (25 µ L, 1:16,000) was immediately added to each well, followed by 25 µl anti-cortisol antibody (1:75,000) (except non-specific binding wells) and incubated at RT for 1 h on a plate shaker set to 150 rpm. Plates were then washed four times with wash buffer (1:20 dilution, 20x Wash Buffer Part No. X007; Arbor Assays, Ann Arbor, MI, USA) and 100 µl of TMB dihydrochloride dissolved in phosphate-citrate buffer with sodium perborate (Sigma Aldrich, St. Louis, MO, USA) was added, followed by incubation for 10 minutes at RT without shaking. The reaction was stopped with 50 µl stop solution (1N HCl), and absorbance was measured at 450 nm by a microplate reader (TECAN Sunrise, Salzburg, Austria). Assay sensitivity based on 90% binding was 0.084 ng/ml. The cortisol EIA was validated for human saliva by demonstrating parallelism between serial dilutions of saliva and the cortisol standards (y =  ‒71.39x +  72.99, R^2^ =  0.95) and significant recovery of cortisol added to low-concentration saliva before analysis (y =  0.88x +  0.05, R^2^ =  0.99). For dog saliva, parallelism between serial dilution of saliva and cortisol standards (y =  ‒133.66x +  72.40, R^2^ =  0.94) and significant recovery of cortisol added to low-concentration saliva before analysis (y =  0.86x +  0.12, R^2^ =  0.99) were observed. Samples were analyzed in duplicate; intra- and inter-assay CVs were 7.2% and < 10%, respectively.

### Fecal processing and glucocorticoid metabolite analysis

Fresh fecal samples (20 g) were collected and placed into zip-lock plastic bags and frozen at ‒20°C until processing and analysis as described [30–32]. Frozen fecal samples were thawed at RT and placed in a conventional oven (60°C) for 24–48 hr. Powdered feces were mixed and ~ 0.1 g (±0.001 g) placed into glass tubes followed by addition of 4.5 ml of ethanol (EtOH) and 0.5 ml of distilled water and vortexing. Samples were extracted twice, first by boiling in a water bath (90°C) for 20 minutes, with 95% EtOH added to keep the volume at 5 ml. Samples were centrifuged at 2,000 x g for 20 minutes and the supernatant decanted into labeled tubes. Another 5 ml of 90% ethanol was added to the pellet, which was then vortexed briefly and centrifuged at 2000 x g for 20 minutes. The fecal extracts were combined and dried in a 90°C water bath. Fecal extracts were re-suspended in 3 ml of 95% EtOH, dried down again, and finally re-suspended in 1 ml of 50% methanol. Samples were stored at ‒20°C until analysis.

Concentrations of fGCM in dog samples were measured in extracts diluted 1:50 in assay buffer using a double-antibody EIA with a polyclonal rabbit anti-corticosterone antibody (CJM006, Coralie Munro, UC California, Davis). Samples and corticosterone standards (50 µl) were added to wells in duplicate, followed by corticosterone-HRP (25 µl; 1:30,000) and then anti-corticosterone antibody (25 µ L; 1:100,000). Plates were incubated at RT for 2 hr before adding 100 µl of TMB solution, followed by incubation for 20–35 minutes and the addition of stop solution (50 µ L). Incubations were conducted in the dark. The absorbance was measured at 450 nm by a microplate reader (TECAN, Männedorf, Switzerland). The corticosterone EIA was validated for dog feces by showing parallelism between serial dilutions of fecal extracts and standards (y = ‒67.71x + 70.04, R^2^ =  0.93) and significant recovery of corticosterone added to a low concentration sample before analysis (y = 1.03x + 0.03, R^2^ =  0.99). Intra- and inter-assay coefficients of variation (CV) were 5.40% and < 10%, respectively.

### Statistical analysis

Data were collected repeatedly from individuals, so a general linear mixed model (GLMM) was used to account for repeated measures and random effects of individuals [33, 34]. The statistical form of the GLMM was:


$$y_{it}=\beta_{0}+\beta_{1}x_{ti}^{\left(1\right)}+\beta_{1}x_{ti}^{\left(2\right)}+...+\beta_{k-1}x_{it}^{\left(k\right)}+u_{i}+\varepsilon_{i}$$

where $y_{i}$ is the response variable (e.g., cortisol, DBP, SBP, PR) from $i^{th}$ individual at time *t*. The terms $\beta_{1}x_{ti}^{\left(1\right)},\beta_{1}x_{ti}^{\left(2\right)}$ and $\beta_{k-1}x_{it}^{\left(k\right)}$ are the fixed effect components of the model (e.g., time, sex and faculty) while $u_{i}$ represent the random effect from $i^{th}$ individual. It was assumed that $u_{i}∼\left(0,D\right)$ and $\varepsilon_{it}∼N\left(0,\sigma^{2}\right)$. Accordingly, the error term $\varepsilon_{it}∼N\left(0,\sigma^{2}\right)$ was used for testing the model assumption including normality and homogeneity of variance by examining normal QQ-plot and plot between $\varepsilon_{it}$ and fitted values.

The residual covariance structures including independent, compound symmetry, autoregressive, and unstructured structures were examined when modelling. The most appropriate structure was justified based the lowest Akaike’s Information Criterion (AIC) value. The model assumptions were not met for the self-reported stress scores, so those data were analyzed using Generalized Estimating Equations (GEE) R version 4.2.3 package geepack, function geeglm. This method accounts for discrete outcomes and repeated measures. Instead of assuming that data were generated from a certain distribution, the GEE uses moment assumption to interactively choose the best estimate to describe the relationship between factors and the response variable [35].

## Results

### University students

**Fig 3:**
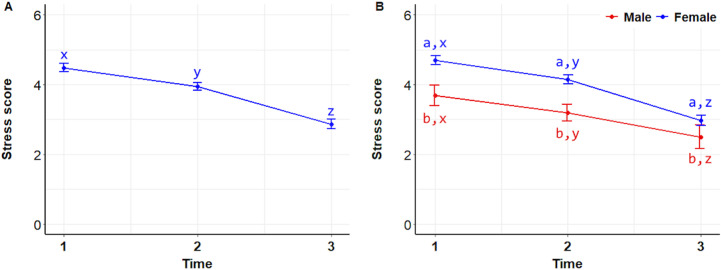
Stress scores. Mean ( ± SEM) stress scores of 122 students during the initial stress assessment (T1), immediately before meeting a dog (T2), and after (T3) interacting with a dog (A), and analyzed by sex (B). Superscripts indicate differences between groups (a, b) and over time (x, y, z).

For all data combined, stress scores after interacting with the dogs (T3) were significantly lower than those before interactions (T1) (P =  0.0017). Additionally, significant differences were found between T1 and T2, as well as between T2 and T3 (P <  0.0001) (Fig 3A). Stress scores decreased similarly over time in both males and females (T1–T2, P =  0.0017; T1–T3, T2–T3, P <  0.0001), but with females reporting higher stress overall than males (P =  0.0001) (Fig 3B). Descriptive statistics for measures evaluated in the study are presented in Table 1.

**Table 1:** Descriptive statistics. Mean ±  standard deviation (SD) values for stress score, systolic blood pressure (SBP), diastolic blood pressure (DBP), pulse rate, and salivary cortisol in university students (n =  122; 30 males, 92 females).

Parameters		Mean ± SD		
		T1	T2	T3
Stress score (1–7)	Overall	4.27 ± 1.319^a,**,^^b,***^	3.73 ± 1.295^b,c,***^	2.66 ± 1.552^a,c^
	Male	3.92 ± 1.476	3.37 ± 1.174	2.30 ± 1.647
	Female	4.63 ± 1.173	4.09 ± 1.247	3.02 ± 1.496
SBP (mmHg)	Overall	116 ± 13.365	113 ± 12.871	113 ± 13.974
	Male	122 ± 13.129	120 ± 10.605	120 ± 13.329
	Female	107 ± 12.109	107 ± 11.993	107 ± 12.752
DBP (mmHg)	Overall	75.8 ± 10.550	74.3 ± 9.955	75.1 ± 9.330
	Male	77.2 ± 10.923	75.7 ± 11.228	76.5 ± 9.991
	Female	74.4 ± 10.343	72.9 ± 9.363	73.7 ± 8.996
Pulse rate (bpm)	Overall	82.2 ± 12.417^d,*^	79.2 ± 11.707	78.6 ± 11.919^d^
	Male	79.3 ± 9.845	76.3 ± 10.932	75.7 ± 11.600
	Female	85.0 ± 12.709	82.1 ± 11.558	81.5 ± 11.820
Salivary cortisol (ng/ml)	Overall	6.08 ± 3.318^e,**^	N/A	5.12 ± 2.180^e^
	Male	6.09 ± 4.482		5.13 ± 1.810
	Female	6.07 ± 2.930		5.11 ± 2.306

SD =  standard deviation, T1 =  before an encounter with a dog, T2 =  immediately before meeting a dog, T3 = after the interaction, SBP =  Systolic Blood Pressure, DBP =  Diastolic Blood Pressure,

*,**,*** = significant at *p* < 0.05, 0.01, 0.001, ^a^–^e^ = significantly different for each pair.

**Fig 4:**
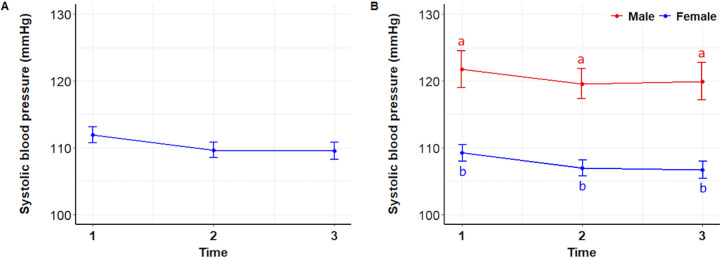
Systolic blood pressure. Mean ( ± SEM) systolic blood pressure of 122 students during the initial stress assessment (T1), immediately before meeting a dog (T2), and after (T3) interacting with a dog (A), and analyzed by sex (B). Superscripts indicate differences between groups over time (a, b) and over time (x, y, z).

For systolic blood pressure (SBP), there were no consistent changes across time periods (P =  0.2962) for all data combined (Fig 4A) or for males and females separately; however, males consistently had higher SBP than females (P <  0.0001) (Fig 4B).

**Fig 5:**
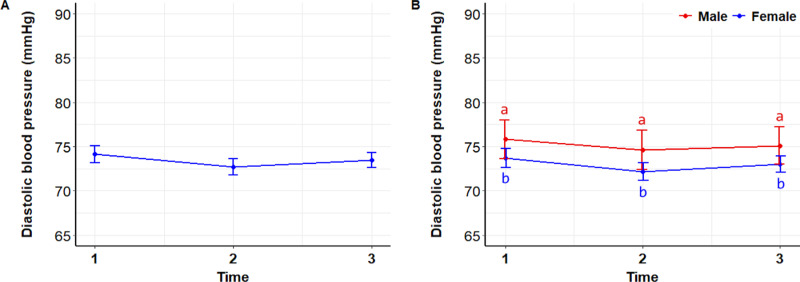
Diastolic blood pressure. Mean ( ± SEM) diastolic blood pressure of 122 students during the initial stress assessment (T1), immediately before meeting a dog (T2), and after (T3) interacting with a dog (A), and analyzed by sex (B). Superscripts indicate differences between groups over time (a, b) and over time (x, y, z).

There were no differences in diastolic blood pressure (DBP) over time (P =  0.541) (Fig 5A), although males had consistently higher DBP than females (P =  0.0324) (Fig 5B)

**Fig 6:**
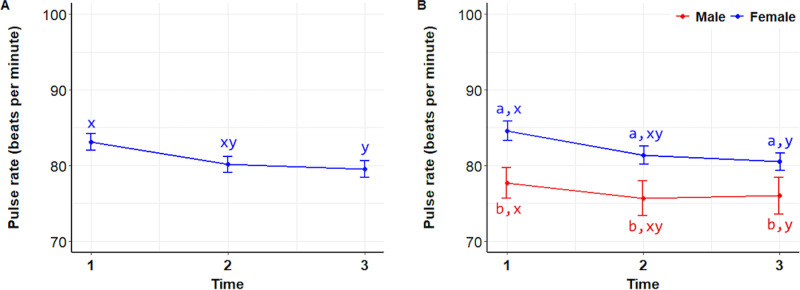
Pulse rates. Mean ( ± SEM) pulse rates of 122 students during the initial stress assessment (T1), immediately before meeting a dog (T2), and after (T3) interacting with a dog (A), and analyzed by sex (B). Superscripts indicate differences between groups over time (a, b) and over time (x, y, z).

Pulse rate (PR) after interacting with a dog (T3) was significantly lower than before the interaction (T1) (P =  0.0406). However, no significant differences were found between T1 and T2 (P = 0.1040) or between T2 and T3 (P = 0.9046) (Fig 6A). When comparing PR between T1 and T3 within sex groups, PR decreased for both males and females after interacting with the dogs (P =  0.0406). However, no significant difference was observed within sex groups at T2 compared to either T1 or T3. Females consistently showed higher PR than males across all time points (P =  0.0001) (Fig 6B).

**Fig 7:**
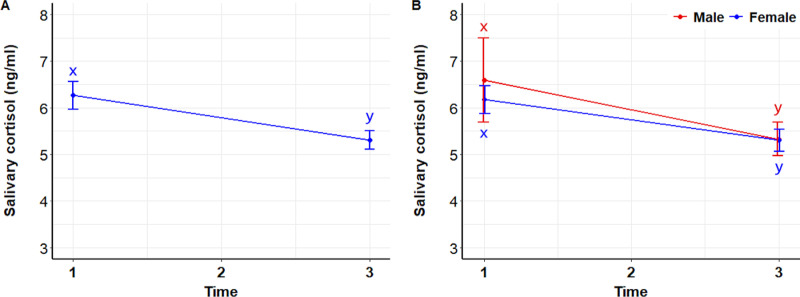
Salivary cortisol. Mean ( ± SEM) salivary cortisol of 122 students during the initial stress assessment (T1), immediately before meeting a dog (T2), and after (T3) interacting with a dog (A), and analyzed by sex (B). Superscripts indicate differences between groups over time (x, y).

Salivary cortisol concentrations after interacting with dogs (T3) were significantly lower than before the interactions (T1) (Fig 7A) in both sexes (P =  0.0081), with no difference between the sexes (P =  0.9614) (Fig 7B).

### Dog salivary cortisol and fGCM

Salivary cortisol concentrations did not differ throughout the day (morning, 5.6 ±  0.8; noon, 3.5 ±  0.6; afternoon, 4.7 ±  0.7 ng/ml). By contrast, fGCM concentrations during the week dogs interacted with students (628.0 ±  24.4 ng/g) were higher than those during the week post-experiment (524.0 ±  18.3 ng/g) (P =  0.0012).

## Discussion

The principal finding of this study underscores how even brief interactions with dogs can significantly reduce stress levels among university undergraduate students based on lower self-reported stress scores. First, compared to the initial time point (T1), stress levels were lower at T2, when participants received information about the dogs - such as their names, personality traits, and preferred interactions - a noteworthy finding in this study. According to social cognitive theory [36], expectations about the consequences of action are learned through observing others. If people observe others being happy from interacting with dogs, they are more likely to feel positive about an upcoming activity. Similar to Vroom (2005) [37], people are driven to behave in specific ways depending on what they believe the results of their actions will be. They expect to feel satisfied if they achieve their desired goal. These ideas are supported by studies that found that optimizing expectations can reduce stress [38,39]. In agreement with those studies, the anticipation of interacting with dogs appeared to lower the self-reported stress levels of our students as well.

Second, compared to immediately before (T2), students’ self-reported stress levels decreased by 33.5% after interacting with the dog (T3). This finding is consistent with a study that, on average, found stress measured by a VAS decreased by 43.1% within 30 minutes of students interacting with dogs [40]. Similar results were found in another study, where students spent an average of 35 minutes interacting with dogs and experienced a 54.8% reduction in stress levels, as measured by a VAS. Other studies have found that interacting with dogs for 15 minutes reduced perceived stress in students by 27.3% and 27.7%, respectively [41,42]. This result may reflect the effectiveness of the CAI for university students in fostering the critical developmental skills of emotion regulation. Pet dogs can serve as companions fulfilling this co-regulatory role, and human-dog interactions encompassing activities like seeing, hearing, touching, talking, playing, or being together can provide moments where humans receive co-regulation support from their canine companions [43–45]. This emotional co-regulation is unique and distinct from human-human interactions during counseling sessions because the dogs can help students feel more at ease and less concerned about being evaluated [46].

Two physiological stress markers, salivary cortisol and pulse rate, were significantly lower when interacting with the dogs (T3) than before encountering them (T1). The salivary cortisol concentrations of students were about 16%, aligning with findings from another study that reported a 31% reduction in salivary cortisol levels after interacting with dogs for 45–60 minutes during exam periods [47]. Similarly, another supporting study found that students who interacted with dogs during a stress-inducing test had lower cortisol concentrations than other groups [48]. Finally, students’ pulse rates were lower after interacting with the dogs than before encountering them, further supporting the stress-reducing effects of canine interactions.

It is possible that merely expecting to meet the dogs may not consistently reduce stress if participants do not engage with the dogs in the way they anticipate. This phenomenon can be explained through two psychological perspectives. First, the anticipation of dogs’ presence may reduce stress by trigger relaxation responses. Second, the entire process involving the dogs can be explained by three key factors: (1) the dogs provide contact comfort; (2) the dogs serve as a pleasant external focus of attention; and (3) participants feel a sense of belonging and support. These factors help participants feel safe and experience pleasure through petting or touching the dogs [49,50]. However, all students in this study responded to dogs with reduced stress levels and associated biomarkers, highlighting that the anticipation of engaging with a dog is another key element of the CAI process. Additionally, although not the primary focus of our study, the period between T1 and T3 may have served as a time when participants experienced a positive distraction from academic pressure, including the actual interaction time with the dogs.

This study also assessed adrenal activity in the dogs. It is well-known that cortisol in diurnal mammals follows a circadian rhythm, being highest in the morning and decreasing until sleep [51]. However, we found salivary cortisol concentrations remained consistent throughout the day, possibly in response to continued playing with students, which kept them stimulated. Significantly higher fGCM concentrations in dogs during the study week compared to the week following interaction sessions again support increased stimulation during play with students. Though not quantified, dogs appeared to enjoy interacting with the students and showed signs of affection and engagement, which suggests that increased adrenal activity and higher cortisol concentrations were due to positive stimulation and not negative stress.

## Conclusions

This study found significant reductions in measures of stress among university students following a brief 15-minute interaction with dogs, aligning with previous research indicating the stress-reducing effects of such interactions. These findings underscore the potential utility of incorporating the dogs into stress management programs within university settings. Adrenal measures in dogs revealed consistent salivary cortisol secretion throughout the day during animal-assisted interventions. By contrast, fGCM concentrations were higher in the week during interactions, suggesting dogs experienced adrenal stimulation when playing with students. Follow-up studies should incorporate additional measures to differentiate between positive stimulation and distress due to these interactions to ensure they reflect dogs enjoying periods of play with students.

Overall, these findings contribute to our understanding of the beneficial impact of human-dog interactions on human stress levels and highlight the importance of addressing stress in both humans and animals during targeted interventions. In contexts where certified dogs and handlers are not readily available, non-certified dogs could still offer mental health benefits. However, establishing objective criteria for dog selection remains challenging. In the future, clearer selection criteria could help produce more specific findings and strengthen the evidence base. We suggest that future studies include multiple sampling time points (e.g., 15-, 30-, and 60-min post-interaction) to characterize the complete cortisol secretory pattern better. Another limitation is that this CAI intervention may have only short-term effects, and thus, it may benefit students to continue therapy on an as-needed basis for ongoing support. There is also an opportunity to explore long-term or continuous sessions to assess its sustained effectiveness. Finally, because our current study lacked a control group, the results should be interpreted with caution.
